# The Dunning–Kruger effect does not account for Covid-scepticism or vaccine hesitancy

**DOI:** 10.1098/rsos.260147

**Published:** 2026-10-07

**Authors:** Robert D. McIntosh, Justin Claydon, Tom Booth, Sergio Della Sala

**Affiliations:** Psychology, The University of Edinburgh, Edinburgh, UK

**Keywords:** Dunning–Kruger effect, vaccines, Covid-19, vaccine-scepticism, vaccine hesitancy, metacognition

## Abstract

Vaccine-scepticism and vaccine hesitancy are sometimes explained using the Dunning–Kruger effect (DKE), which posits that individuals with low knowledge in relevant domains are overconfident in their understanding. However, prior evidence often relies on discrepancy-based measures of self-overestimation that are susceptible to statistical artefacts. It, therefore, remains unclear whether vaccine-scepticism genuinely reflects the defining features of the DKE. During the Covid-19 pandemic, a large UK sample (*n* = 537) completed a newly developed Covid-scepticism (CS) scale, with a knowledge test assessing biological knowledge and probabilistic reasoning, relevant to a viral pandemic. Item-level confidence ratings for each knowledge judgement were used to derive a measure of metacognitive bias, which indexes a person's overall tendency towards low or high confidence. Higher CS was associated with lower levels of knowledge. The relationship with low knowledge was strongest for sceptical attitudes towards vaccines and endorsement of conspiracy-adjacent beliefs. However, lower knowledge was not associated with overconfidence, as predicted by DKE accounts. Instead, metacognitive bias increased with knowledge, and higher vaccine-scepticism predicted lower overall confidence. These findings provide little support for accounts of vaccine-scepticism based primarily on the popular view of the DKE. The results underscore the importance of using appropriate measures of metacognition and caution against dismissive characterizations of vaccine-sceptic attitudes.

## 1. Introduction

### 1.1. The Dunning–Kruger effect as an account for vaccine-scepticism

In Plato's cave allegory (375 BC), prisoners are confined to a dark cave, seeing only shadows cast on a wall, their distorted perceptions of reality. This symbolizes how people can be trapped in ignorance, mistaking their limited knowledge for the whole truth. This archetype epitomizes a common human foible: the tendency to overestimate our abilities while remaining blind to our shortcomings. Deficiency in self-awareness of one's own abilities has been deplored by philosophers, writers and scientists throughout history and across geographical areas [1]. Within the psychology literature, the tendency to judge oneself as more knowledgeable or skilful than others has been branded variously: *illusory superiority* [2], *sense of relative superiority* [3] and the *better-than-average effect* [4].

A closely related phenomenon is the *Dunning–Kruger effect* (DKE; [5]), named after the authors of the paper which reported it [6]. The DKE describes a situation in which individuals with poor knowledge or skill in a topic or domain overestimate their competence, either relative to others or in absolute terms, in contrast to those with higher knowledge levels, who tend to be more accurate in their self-assessments (albeit they may slightly underestimate how much better than their peers they are). This pattern is often said to arise because less knowledgeable individuals lack the self-awareness to recognize their own deficiencies, while more skilled individuals have better self-insight. The effect underscores the importance of metacognitive skills, that is understanding one's own knowledge and abilities, in shaping accurate self-assessment.

In recent years, the DKE has become a popular explanation to account for the lack of compliance with national health policies and has been invoked to explain the behaviour of vaccine-hesitant people. The argument is that people who have only a superficial understanding of vaccines would dismiss scientific consensus and public health recommendations as they feel overly confident in their beliefs, based on anecdotal evidence or misinformation. The phenomenon of vaccine hesitancy became particularly prominent during the Covid-19 pandemic, and the DKE as a short cut explanation spread like a virus: people are sceptical of vaccines because they are ignorant of the topic and do not acknowledge their ignorance.

At the same time, people who expressed Covid-scepticism (CS), especially by flaunting official guidance around coronavirus, were branded in the popular press as ‘Covidiots’, a pejorative neologism that implies low intelligence or ignorance (e.g. [7]). Pre-pandemic, the same narrative was applied to people who were sceptical about other vaccinations, both in news outlets (e.g. [8]) and in the academic literature [9]. These caricatures are based on rather weak evidence, and they risk creating divisive debates, which hamper dialogue and a deeper understanding of the attitudes involved. The main purpose of this study is to test critically whether there is an empirical basis for portrayals of vaccine-sceptic people as ignorant of relevant information and overconfident in their level of knowledge. To address this question, we capitalized on the specific phenomenon of CS during the Covid-19 pandemic.

### 1.2. Knowledge, confidence and the Dunning–Kruger effect

To form rational attitudes about coronavirus and to accurately appraise the value of mitigation measures, some level of scientific literacy should be important. This includes an awareness of some basic biological science and an ability to reason probabilistically. In a two-wave study across the United States (US), Canada and the UK, Pennycook and colleagues [10] measured CS in terms of Covid-misperceptions and Covid-risk perceptions using a ‘cognitive sophistication’ scale, which combined scientific knowledge, a cognitive reflection test of analytical thinking [11], probabilistic reasoning and susceptibility to pseudo-profound bullshit [10]. They found that both measures were robustly associated with reduced mitigation behaviours and vaccination intentions. Teovanović and colleagues [12] similarly used the cognitive reflection test [11] as a measure of ‘cognitive intuition’ and probabilistic reasoning problems, both of which correlated with Covid-conspiracy thinking. Expressions of CS may thus be related to lower knowledge of relevant science and/or to less analytical reasoning.

Teovanović *et al.* [12] adopted a method from the literature on the DKE, whereby the discrepancy between a person's self-rated ability (e.g. self-estimated test score) and their actual ability (estimated from an objective test score) is taken to index how much they under- or overestimate themselves [6]. Across many domains, those with the poorest objective scores show the greatest levels of self-overestimation (see [5] for a review of early findings). Such self-overestimation is common in matters of medical knowledge and assessment of one's own health risks (see [13]). It has also been shown to apply to the analytical reasoning skills tapped by the cognitive reflection test: people scoring lowest on this test grossly overestimate their reasoning skills, while those scoring highest slightly underestimate them [14]. Teovanović and colleagues [12] hypothesized that overconfidence in one's knowledge of the coronavirus could lead people to ignore official guidance on risks, mitigations and vaccination. Although their measure of knowledge overestimation did correlate significantly with reduced adherence to Covid guidelines (*r* = −0.12), the association was too weak to robustly support their hypothesis.

One reason for the weakness of the observed association could be that the measure of knowledge overestimation was insufficient. It was based on the discrepancy between (normalized) scores of self-estimated and actual knowledge, where the self-estimate was a single Likert scale rating of one's own knowledge of the virus, while actual knowledge was inferred from nine true/false statements (e.g. ‘The coronavirus is spread by respiratory droplets’). Given that approximately 50% correct answers could be expected by chance, this nine-item knowledge test has little scope to distinguish true knowledge from guesswork, and the discrepancy of this score from a single self-estimate will be even more noisy. This is a microcosm of a broader issue that the rush to study psychological attitudes to an unexpected and fluid pandemic in real time often meant the adoption of novel, ad hoc measurement scales, with relatively little attention paid to their psychometric properties.

There are also more fundamental problems with discrepancy-based measures of self-overestimation [15]. When we subtract an objective ability score from a self-estimated ability score, the discrepancy is almost guaranteed to correlate negatively with objective ability, because the objective ability score has entered negatively into the calculation of the discrepancy (we have spuriously correlated X with Y minus X). When using such discrepancy scores, we should never be surprised to find that the poorest performers overestimate themselves the most. Imagine that no one had any insight at all into their own ability and rated themselves by default at 3 or 4 on a five-point scale. This would be a major overestimate for the least able performers and a minor underestimate for the most able, but we would be wrong to conclude that they had differing underlying levels of insight. The pattern would instead be a regression-to-the-mean artefact [16]. The central role of statistical artefacts in shaping the classic DKE has been discussed extensively in the academic literature [17–23] and debated in scientific magazines [1,24,25]. The critical point is that discrepancy scores are not meaningful measures of self-insight, and we should not use them.

Given growing awareness of fundamental problems with discrepancy-based measures of metacognitive insight, some researchers have applied more sophisticated analyses, for instance testing for nonlinear components in the relationship between self-assessed and objective ability that cannot be dismissed as statistical artefacts [26–29]. Some of these analyses do suggest genuine metacognitive miscalibration among poor performers, but the biases identified are negligible in practical terms, affecting only a small proportion of extreme cases [30], and cannot sustain an explanatory role for the DKE in large-scale behaviour. A different approach has been to derive rigorous psychophysical measures of metacognitive insight from trial-by-trial confidence ratings during simple motor and memory tasks [22] or more complex logical reasoning [31]. These studies found reduced metacognitive sensitivity among poor performers, who were less able to distinguish their successful responses from their errors, but no evidence that this led them to overestimate their abilities; indeed, poor performers expressed appropriately lower confidence than their more skilled counterparts. Despite broader methodological advances in the metacognition literature [32,33], flawed discrepancy-based measures of self-estimation have persisted and have been widely deployed in support of the claim that overconfidence in one's own knowledge underlies opposition to Covid vaccination and other preventative measures [34–42]. These claims have been further amplified through opinion pieces and editorials in medical journals [43,44], as well as in the popular press and online.

One study on Covid provides a clear illustration of why we should not use these measures. Claessens and colleagues [45] had 2043 hospital visitors complete a multiple-choice scientific quiz about Covid-19 and then rate their own knowledge of Covid out of 10. Performance on the quiz was quite poor, averaging around 35% for the bottom quartile and 55% for the top quartile, but the mean self-estimate was close to 7 out of 10 (i.e. 70%) across the ability range. This was a gross overestimate for the poorest performers and a more accurate estimate for the best performers, so the DKE narrative was supported. However, it is vital to note that the self-rating was completely unrelated to knowledge level, suggesting that people, in general, had no real sense of how good their knowledge of Covid was but just defaulted to a guess of around 7 out of 10 on average. Subtracting their knowledge score from this constant self-rating then produced the classic DKE pattern as a statistical artefact.

A further point to note is that, for each item in the knowledge quiz, participants were also asked to rate, on a four-point scale, how certain they were. The poorest performers on the knowledge quiz expressed considerably lower certainty than the top performers. This pattern suggests that the least knowledgeable people did not genuinely overestimate their knowledge, because they were appropriately unconfident about their answers. If we want to draw meaningful conclusions about a person's insight into their knowledge or abilities, then rather than asking them to estimate their total score, we may be better to follow the strategy of asking them how confident they feel in each of their answers.

### 1.3. The avoidance of circularity

The use of confounded discrepancy-based measures of self-overestimation is not the only pitfall in this general area. If the aim is to study the relationship between attitudes and the level of knowledge relevant to that issue, we must avoid direct overlap in the measurement of attitudes and knowledge, or tautological conclusions can follow. There are cautionary examples to be found in the wider literature on vaccine-scepticism. For instance, Motta and colleagues [9] reported that people opposed to measles, mumps, rubella (MMR) vaccination tended to know the least about autism. However, the test of autism knowledge included an item about the (discredited) link between the MMR vaccine and autism, and it is self-evident that anyone failing this item would be likely to oppose the MMR vaccine. This study effectively measured the same thing twice, once in the name of knowledge and once in the name of attitudes, promoting a spurious link between them. Another study found that anti-vaccination attitudes were linked with a tendency to overestimate the risk of rare negative events [46], but the rare negative events that participants were asked about included the rates of US deaths from smallpox and measles. It is unsurprising to learn that people who overrate the risks of diseases that have been eradicated by vaccination should also express less pro-vaccination attitudes. Such methodological circularities, leading to uninformative conclusions, need to be avoided.

### 1.4. The present study

The aim of the present study was to test the association between scepticism of public health information, a lack of knowledge in relevant domains and an excess of confidence about one's own level of knowledge, steering clear of the pitfalls identified above. To this end, we capitalized on the opportunities presented by the Covid-19 pandemic, after Covid vaccines have been approved for public use in the UK and US.

We first developed a novel scale to measure CS. Since this is neither a unitary nor a formally defined construct, we aimed to capture a range of salient themes, drawing on mainstream media and online sources. In particular, item content was informed by a website (*Anti-virus: The Covid-19 FAQ*) created by a group of academics, journalists and volunteers to identify and counteract misinformation about Covid-19. The item-generation and scale-development process is described in detail in the supplementary material; the final scale is described in the ‘Methods’. We also designed a pandemic-relevant knowledge test, following Pennycook *et al.* [10] in assessing scientific knowledge and probabilistic reasoning of relevance to making rational decisions about Covid, while avoiding circular overlap with the items assessing CS. We did not infer people's confidence in their knowledge from discrepancy-based measures of self-estimation but instead collected item-level ratings of confidence during the knowledge test. The knowledge test does not meet the restrictive set of assumptions required for computational modelling of metacognitive sensitivity and efficiency, but we are able to extract a simpler measure known as metacognitive bias, which is the unweighted average confidence rating across correct and incorrect answers [15]. This metacognitive bias measure reflects a person's tendency towards high or low confidence independent of their level of performance, which would support more secure inferences about whether Covid-sceptic people are overconfident in their knowledge. We administered these tests to a large sample of participants online, to evaluate whether CS is associated with a lack of knowledge in relevant domains, and to test the pattern predicted by the DKE, that people with less knowledge will be overconfident about their knowledge.

## 2. Methods

### 2.1. Preliminary scale development

The preliminary stages of this project focused on the psychometric development of a scale for CS and a test of domain-relevant knowledge. The Covid-scepticism (CS) scale development involved online data collection from a sample of 357 adults in the UK and US between 5 July and 4 August 2021 and is described in the supplementary material S1. The knowledge test development involved online data collection from a sample of 99 adult participants in the UK and US between 12 and 14 October 2021 and is described in the supplementary material S2.

### 2.2. Participants

For the main study, 563 adult participants were recruited online from Prolific Academic (http://prolific.co/) between 19 and 24 January 2022. The sample was requested to be representative of the UK adult population, for sex, age and ethnicity (according to census data). Sample size was determined as the largest number that resources allowed. Participants viewed an information page, before indicating consent and proceeding to the survey. The survey took a median time of 13 minutes and 21 seconds to complete, and participants were compensated at a standard rate for completion. The study was approved by the PPLS Research Ethics Committee at the University of Edinburgh.

The final sample (after all exclusions; see §2.4, ‘Data Processing’) had 537 participants, the demographic details for whom were provided by Prolific. Participants had the option to withhold demographic data, and the *n* values below indicate the size of the subsample that each summary is based on. There were 52% women and 48% men (*n* = 534), with a mean age of 45.5, s.d. 15.8 (*n* = 534), and English was the first language for 91% participants (*n* = 531). All participants answered the question about Covid vaccination status: 498 (93%) reported having received a vaccine, 24 (4%) reported that they would not accept if offered, and 15 (3%) were unsure.

### 2.3. Material and procedure

The online survey, created and administered using Qualtrics (http://www.qualtrics.com), included the following components, in order: CS scale; knowledge test; general conspiracy beliefs (GCB) scale; additional questions. Each component is described below.

#### 2.3.1. Covid-scepticism scale

The *CS scale* comprised 46 statements expressing attitudes about various aspects of the Covid-19 pandemic. As shown in table 1, the 46 statements represented 23 conceptual items, with each conceptual item composed of two opposed statements, one expressing a Covid-orthodox (CO) attitude and the other expressing a CS attitude. CO statements reflect mainstream narratives about the pandemic, and CS statements reject those narratives or reflect alternative narratives. The 23 conceptual items were themed around three factors, defined by exploratory factor analysis during scale development. We have construed these three factors as follows: ‘severity/policy scepticism’ (i.e. scepticism that government policies to reduce the spread of the virus are proportionate to the severity of the risk); ‘vaccine/conspiracy scepticism’ (i.e. scepticism about the effectiveness and safety of Covid vaccines, and endorsement of pandemic-related conspiracy theories); and ‘health/mechanism scepticism’ (i.e. scepticism about mainstream messages about how the virus is transmitted and the health problems it causes).

**Table 1.** The Covid-sceptic form of the statements for the 23 conceptual items in the CS scale, classified according to three latent factors: severity/policy scepticism (SPS), vaccine/conspiracy scepticism (VCS) and health/mechanism scepticism (HMS). (Each item also included a CO form of the statement, for a total of 72 statements. For instance, the CO statement for item 1 was ‘Covid-19 vaccines do not contain technology that allows people to be tracked.’ The CO statements for every item are included in the supplementary material, table S1.1.)

factor	item	statement
VCS	1	Covid-19 vaccines contain technology that allows people to be tracked
VCS	2	patents for Covid-19 vaccines were filed before the outbreak of the pandemic
VCS	3	the Bill and Melinda Gates Foundation owned a patent for a Covid-19 vaccination prior to the outbreak of Covid-19
VCS	4	Covid-19 vaccines can alter a person's DNA
HMS	5	Covid-19 cannot be transmitted by asymptomatic people
HMS	6	Covid-19 is not spread by liquid droplets in the air (aerosols)
HMS	7	long-term after-effects of Covid-19 do not exist (‘long Covid’)
HMS	8	Covid-19 does not spread more easily indoors
SPS	9	quarantine is an unnecessary response to Covid-19
SPS	10	lockdowns do not reduce the spread of Covid-19
SPS	11	measures to reduce the spread of Covid-19 are not needed to protect the health service
SPS	12	governments should not be able to impose restrictions on personal freedom in order to combat Covid-19
SPS	13	the infection fatality ratio of Covid-19 is low enough that we should not be concerned
SPS	14	Covid-19 is not serious enough to justify the measures being taken across the world
VCS	15	cheap medical treatments like vitamin D and ivermectin would prevent or treat Covid-19 effectively
SPS	16	masks do not reduce the spread of Covid-19
SPS	17	social distancing does not reduce the spread of Covid-19
VCS	18	vaccines cannot reduce the symptoms and transmission of Covid-19
VCS	19	vaccines for Covid-19 have caused an increase in care home deaths
VCS	20	Covid-19 vaccines are unsafe
VCS	21	Covid-19 vaccines are ineffective
VCS	22	the fact that there is ongoing monitoring of Covid-19 vaccines indicates they are unsafe
VCS	23	clinical trials of the approved Covid-19 vaccines are insufficiently conclusive

The scale was introduced by these instructions: ‘You will see statements about the Covid-19 pandemic. Your task will be to indicate on a five-point scale whether you think each statement is true/false and how certain you are. Each question has a visible countdown. Once the countdown reaches zero, you will be moved to the next question. Please read every item carefully and try to respond within 20 seconds. If you respond within the time limit, you can press the arrow to proceed to the next question. Some questions may be similar to, or exactly opposite to some other questions. Read each question carefully before you answer.' For each statement, participants indicated their level of agreement on a five-point scale, labelled (from left to right) as follows: definitely false, probably false, not sure/cannot decide, probably true and definitely true. The 46 statements were presented in a random order along with one attention check item (‘It is important that you consider each question and respond accurately. Please click on “Definitely true”’). If participants had not selected a response to any question before the timer reached zero, they were automatically moved to the next question and no response was recorded.

**Table 2.** The 30 questions in the knowledge test, as reduced from the 48 candidate items used in piloting (supplementary material, table S1.6). (The correct response was true for 15 items and false for 15.)

item	question	T/F
Q01	the reproduction number (R) of an infectious disease can change	T
Q02	the word ‘vaccine’ comes from a Latin word relating to cows	T
Q03	a mutation is a change in DNA	T
Q04	SARS is caused by a virus	T
Q05	RNA stands for ribonucleic acid	T
Q06	the largest known virus is 1 mm in diameter	F
Q07	there is a vaccine for yellow fever	T
Q08	the bubonic plague (a.k.a. ‘Black Death’) is caused by a bacterium	T
Q09	viruses can reproduce on unattended surfaces	F
Q10	a disease vector is any agent which carries and transmits an infectious pathogen into another living organism	T
Q11	zoonotic diseases are diseases that have jumped from an animal to a human	T
Q12	the incubation period is the time between symptom onset and recovery	F
Q13	using microscopes, viruses were discovered before bacteria	F
Q14	World War I had a bigger death toll than the 1918 influenza pandemic (a.k.a. the Spanish flu)	F
Q15	antibiotics can kill viruses	F
Q16	red blood cells are part of the immune system	F
Q17	vaccines stimulate the body to produce more red blood cells	F
Q18	viruses can only infect humans and other animals	F
Q19	Zika is a virus spread by mosquitos	T
Q20	hepatitis is caused by viruses	T
Q21	all viruses that infect humans are harmful	F
Q22	the human papillomavirus (HPV) is sexually transmitted	T
Q23	logistic growth describes an S-shaped growing curve	T
Q24	the infection rate is the total number of cases of a disease in a population	F
Q25	if I toss a coin and it lands on tails four times in a row, there is an 80% chance that it will land on heads on the next throw	F
Q26	if 60% of people living in City C get the Virus V, then living in City C is a risk factor for Virus V infection	F
Q27	if a lottery draw has 10 prize tickets and 40 blanks, then the probability of getting a prize with one ticket is 1/5	T
Q28	the inverse of an exponential function is a logarithmic function	T
Q29	viruses reproduce by splitting to produce two identical copies	F
Q30	vaccines work on homoeopathic principles	F

#### 2.3.2. Knowledge test and confidence ratings

The test of domain-relevant knowledge comprised 30 questions assessing biological and historical knowledge about viruses, as well as basic probabilistic reasoning. The questions are listed in table 2. The participant responded to each item by clicking one of two buttons, marked TRUE and FALSE, and was then presented with a screen that asked, ‘*How confident are you in your response*’, selecting from the options: ‘*Not at all confident; A little confident; Mostly confident; Very confident*’. The 30 statements appeared in a random order, with one simple attention check item (‘*To demonstrate that you understand this instruction fully, please choose “Probably false”*’).

#### 2.3.3. General conspiracy beliefs scale

The third part of the survey comprised the 14 questions of the GCB scale, taken from Brotherton *et al.* [47], with a simple attention check question included (as question 6 out of 15). The scale was introduced by these instructions: ‘*There is often debate about whether or not the public is told the whole truth about various important issues. This short survey is designed to assess your beliefs about some of these subjects. Please indicate the degree to which you believe each statement is likely to be true.*’ Each statement was presented on a single page with no timer, and the participant was required to indicate their level of agreement on a five-point scale, labelled (from left to right) as follows: definitely not true, probably not true, not sure/cannot decide, probably true and definitely true. Note that the wording ‘*not true*’ (rather than ‘*false*’) was used to match the wording of the standard version [47]. The order of questions also matched that of the version, with one simple attention check inserted between questions 5 and 6 (‘*It is important you consider each question and respond accurately. Please click on “Definitely not true”*’).

#### 2.3.4. Further questions

The final section of the survey comprised three to four questions. The first question was ‘*Have you received your Covid-19 vaccine already?*’ (yes, no). If the participant responded negatively, they were presented with the second question: ‘*Will you accept a Covid-19 vaccine if offered one?*’ (yes, no, unsure). The participant was then asked to indicate their highest level of education completed (GCSE, A-level, Undergraduate, Masters, PhD) and preferred source of news media (social media, print newspapers, television, radio and news websites).

### 2.4. Data processing and scoring

#### 2.4.1. Participant exclusions

Of the 563 participants who took part, 11 did not finish the survey, none failed more than one attention check, 10 were excluded for discrepant responding (> 10% of responses discrepant) in the CS scale, four were excluded for answering fewer than 28 (i.e. 90%) of the 30 questions in the knowledge test, and one was excluded for scoring at ceiling or floor level on the knowledge test (they scored at ceiling), leaving a final valid sample size of 537. All exclusion criteria were defined in advance.

#### 2.4.2. Covid-scepticism scale scoring

For each participant, for each of the 46 statements (two statements for each of 23 items), the strength of endorsement was converted to a numerical score, such that definitely false = −1, probably false = −0.5, not sure/cannot decide = 0, probably true = 0.5 and definitely true = 1. The scores for the CO statements were then flipped (multiplied by −1) so that CO attitudes were always negative and CS attitudes positive. The pairs of responses to the opposing CO and CS statements per item were checked for discrepancies, defined as a difference greater than one (i.e. more than two notches on the Likert scale). Discrepant items were excluded (coded as missing), and participants were excluded if they had discrepant responses to more than 10% of items (i.e. more than two items); 10 participants were excluded on this basis. Responses to the two statements were then averaged to produce a mean response for each participant for each of the 23 items, where −1 represents maximum CO and +1 represents maximum CS attitudes. The item-level responses per participant were averaged for each of the three factors: severity/policy scepticism, vaccine/conspiracy scepticism and health/mechanism scepticism. The average of the three-factor scores per participant gave their overall CS score.

#### 2.4.3. Knowledge test and confidence ratings scoring

The participant's score for the knowledge test was the number of correct answers (from 30), expressed as a percentage. The second-order confidence ratings in their answers were summarized in different ways. Overall confidence was calculated as the average rating across all answers. The same calculation was then done separately for correct answers (confidence-correct) and for incorrect answers (confidence-incorrect). Confidence-correct and confidence-incorrect scores were then averaged to provide a confidence measure that is unweighted by the rate of correct responding. This final measure, called metacognitive bias, represents the participant's tendency to express low or high confidence, independent of their level of performance on the primary (knowledge) task [15].

#### 2.4.4. General conspiracy beliefs scale scoring

Rather than following the original 5–45 point scale of Brotherton *et al.* [47], we scored the GCB in a manner consistent with the scoring of our CS scale. That is, the Likert scale responses were converted to numerical scores, such that definitely not true = −1; probably not true = −0.5; not sure/cannot decide = 0; probably true = 0.5; definitely true = 1. The average score across the 15 items of the GCB thus produced a final score between -1 and +1, where positive values represent endorsement of conspiracy beliefs. Note that this is a simple linear transformation of the score on the original 5–45 scale.

#### 2.4.5. Covid vaccine hesitancy

Covid vaccine hesitancy was encoded as a binary variable (0, 1), based on responses to the vaccine question. Participants were scored as not hesitant (0) if they reported that they had received a Covid vaccine or that they would accept when offered; otherwise, they were scored as hesitant (1).

## 3. Results

### 3.1. Characteristics of Covid-scepticism scores

As illustrated in figure 1a–c, the three CS latent subfactors each had a positively skewed distribution of scores. This was most marked for the health/mechanism scepticism factor, in which the modal response was at a maximum level of CO (−1). As the total CS score (figure 1d) was the average of the sub-scores, it was also positively skewed (mode −0.74, median −0.73, mean −0.63), with only a small minority of people scoring positively (21 people or 4% of the sample), only two people scoring above 0.5 and not even a small subgroup at the extreme CS end of the scale. It is thus important to bear in mind that the sample was overwhelmingly on the orthodox side, with CS measured as a relative bias towards more positive scores. Given that the sampling strategy was representative, with respect to census data, this outcome implies that orthodox attitudes were similarly dominant within the UK population, with CS measured as a *relative bias towards more positive scores*. The three latent subfactors correlated substantially with one another, with *r* between 0.57 and 0.64 (Spearman correlations were closely similar in all cases). These patterns are similar to those observed in the pilot study during scale development (supplementary material S1.7, figure S1.1).

**Figure 1.**
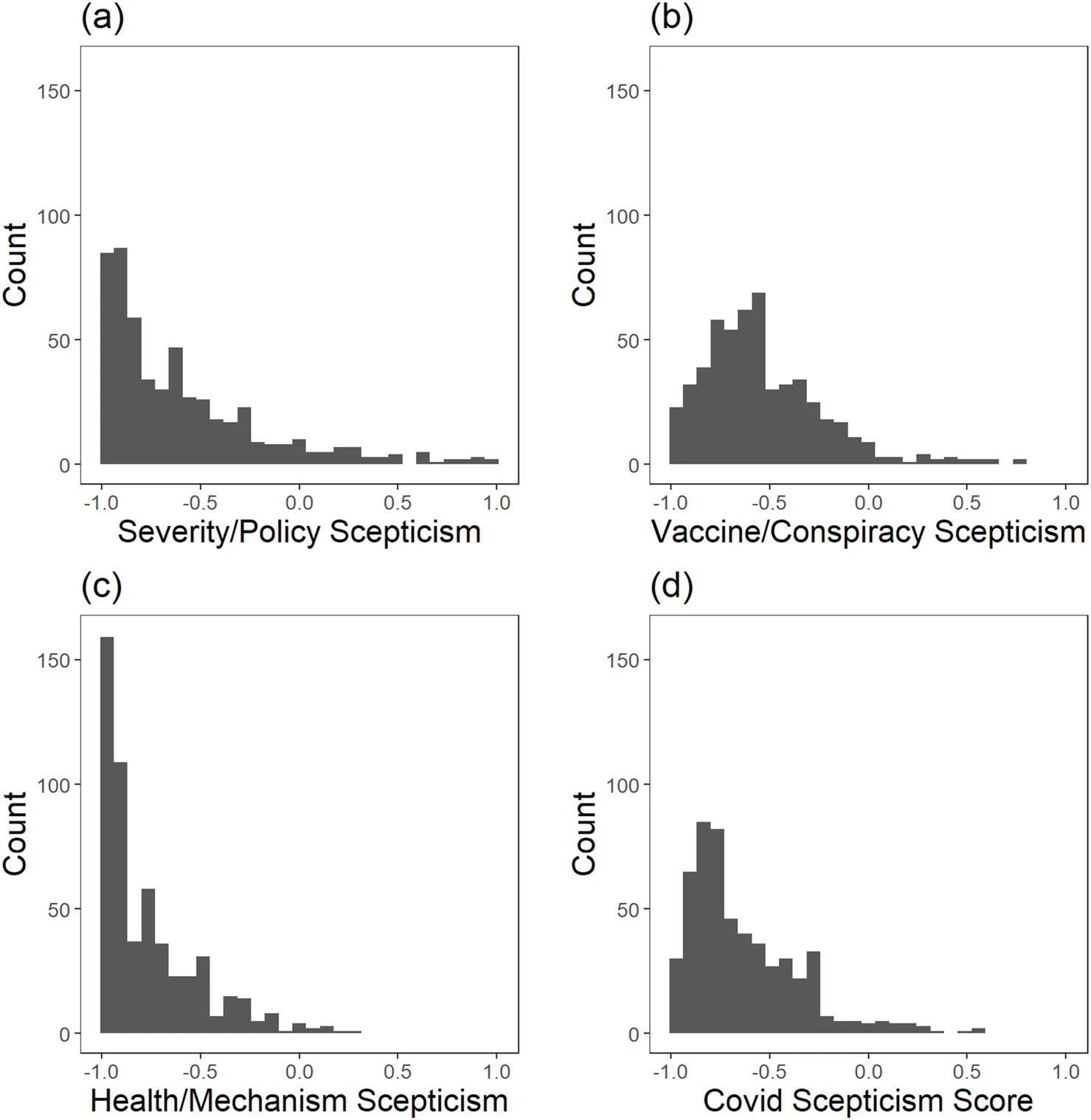
Distribution of scores for the Covid-scepticism (CS) latent subfactors (a–c), and for the total CS score (d).

### 3.2. External validity of Covid-scepticism scale

We were able to check the CS scale for external validity with respect to conspiracy thinking (as measured by the GCB scale) and Covid vaccine hesitancy (as measured by the further questions about vaccinations). These measures are most directly relevant to the vaccine/conspiracy factor; and, unsurprisingly, the vaccine/conspiracy sub-score correlated most strongly with these items, although positive correlations (with confidence intervals (CIs) not including zero) were observed for all factors and for the overall CS score (table 3). These validation outcomes are similar to those observed in the pilot study during scale development (supplementary material S1.7, table S1.3).

**Table 3.** Correlations (and bootstrapped 95% CIs) of factor sub-scores and overall CS score with general conspiracy beliefs and Covid vaccine hesitancy. (The latter is a binary classification, so the correlations in the right column are point-biserial.)

	general conspiracy beliefs	Covid vaccine hesitancy
**severity/policy scepticism**	0.41 [0.33, 0.49]	0.45 [0.34, 0.55]
**vaccine/conspiracy scepticism**	0.62 [0.56, 0.66]	0.59 [0.49, 0.66]
**health/mechanism scepticism**	0.41 [0.33, 0.47]	0.31 [0.19, 0.41]
**CS score**	0.55 [0.50, 0.61]	0.53 [0.43, 0.61]

### 3.3. Is Covid-scepticism associated with a lack of knowledge?

The association of CS with domain-relevant knowledge is shown in table 4 and figure 2. A linear model to predict knowledge score from the three CS sub-scores captured 12% of the variance, but with a slightly unexpected pattern of regression coefficients. Vaccine/conspiracy scepticism and health/mechanism scepticism showed the expected negative relationships, with higher scepticism predicting lower knowledge, but severity/policy scepticism had a weakly positive coefficient. Although the three sub-scores were intercorrelated (*r* = 0.57–0.64), collinearity diagnostics did not suggest any problematic multicollinearity (maximum variance inflation factor = 1.91).

**Table 4.** Coefficients for prediction of domain-relevant knowledge from CS latent factors, with adjusted *R*-squared for each model. (All *p*-values are below the 0.005 level. SPS, severity/policy scepticism; VCS, vaccine/conspiracy scepticism; HMS, health/mechanism scepticism; CSS, CS score (total score).)

	model				
predictor	all sub-scores	SPS	VCS	HMS	CSS
SPS	5.30	−4.50	–	–	–
VCS	−12.98	–	−13.11	–	–
HMS	−10.06	–	–	−14.15	–
CSS	–	–	–	–	−12.50
adjusted *R*-squared	0.12	0.02	0.10	0.07	0.07

**Figure 2.**
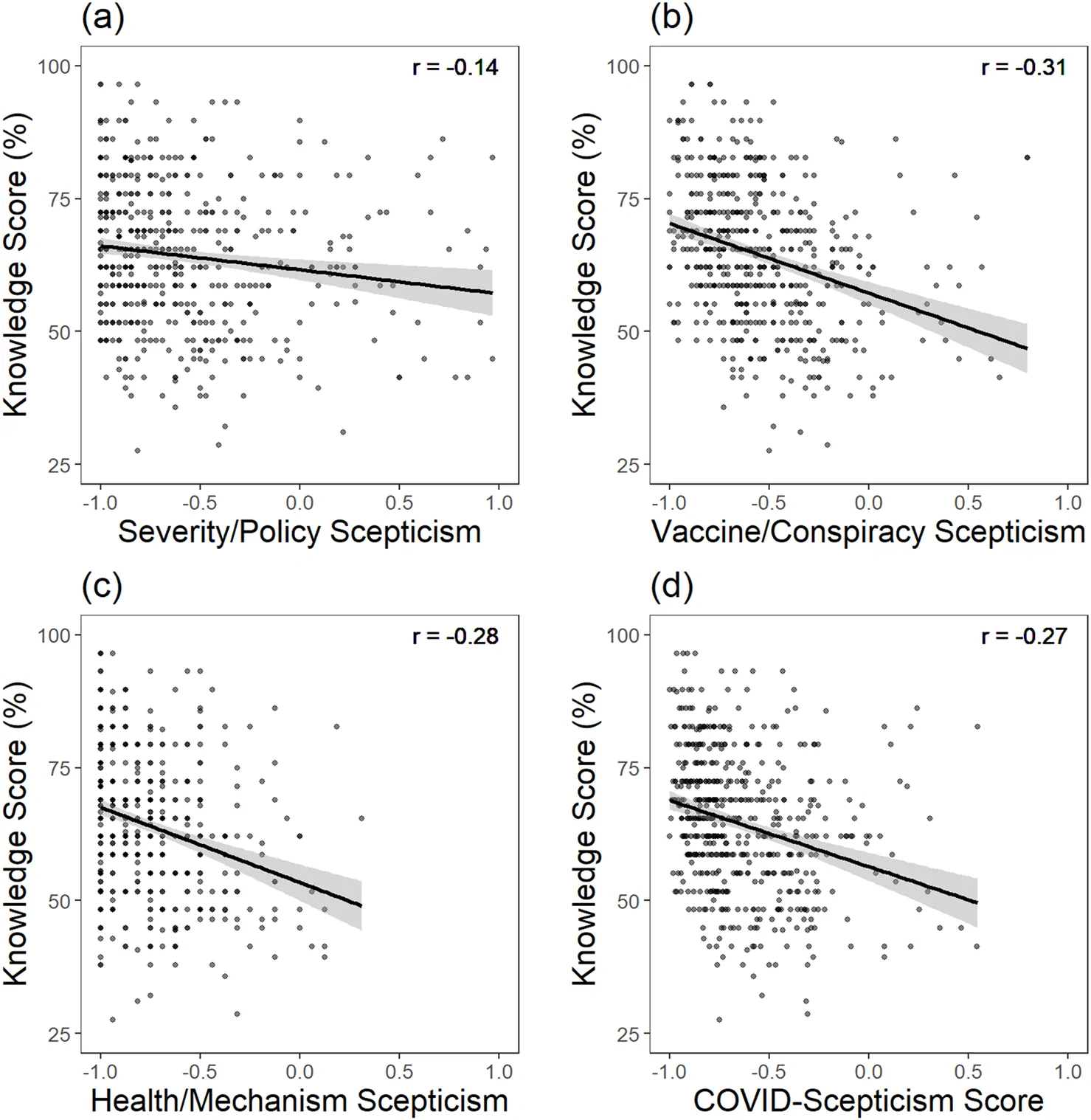
Bivariate relationships for prediction of domain-relevant knowledge from Covid-scepticism (CS) latent factors, and total CS score. Panels (a)-(d) show relationships between domain-relevant knowledge score and severity/policy scepticism, vaccine/conspiracy scepticism, health/mechanism scepticism, and total CS score, respectively, with adjusted R-squared for each model. (All *p*-values are below the 0.005 level.) Shaded areas show 95% CIs around the line of best fit.

To explore this further, we ran separate models to predict knowledge score from each sub-score individually. These single-factor models showed directionally consistent negative effects across scepticism sub-scores (table 4). The sign reversal for severity/policy scepticism is consistent with a suppression effect, whereby its positive coefficient in the three-factor model reflects a unique association with knowledge score after accounting for variance shared with the other predictors, whereas the single-factor model captures the overall negative association between severity/policy scepticism and knowledge score. A final model predicting knowledge score from the total CS score similarly showed a moderate negative relationship, with a one-point increase in CS predicting a 12.5 percentage-point decrease in domain-relevant knowledge.

### 3.4. How does knowledge relate to confidence?

Confidence for the knowledge test is summarized by metacognitive bias, which is the mean of the confidence ratings given for correct answers, and those given for incorrect answers. This measure represents the overall level of confidence expressed, independent of the proportions of correct and incorrect answers. As shown in figure 3a, metacognitive bias nonetheless changes with first-order performance, showing a positive relationship with knowledge score (*r* = 0.26, *p* = 1.38 × 10^−9^). Overall, the lowest-observed level of performance on the knowledge test (28%) predicts a metacognitive bias of 2.0, while the highest observed (sub-ceiling) level of knowledge (97%) predicts a metacognitive bias of 2.7, just above the half-way point on the confidence scale. The data regress towards the middle of the confidence scale overall, but within this context, those with less knowledge are relatively less confident, even after taking account of their higher proportion of incorrect answers.

**Figure 3.**
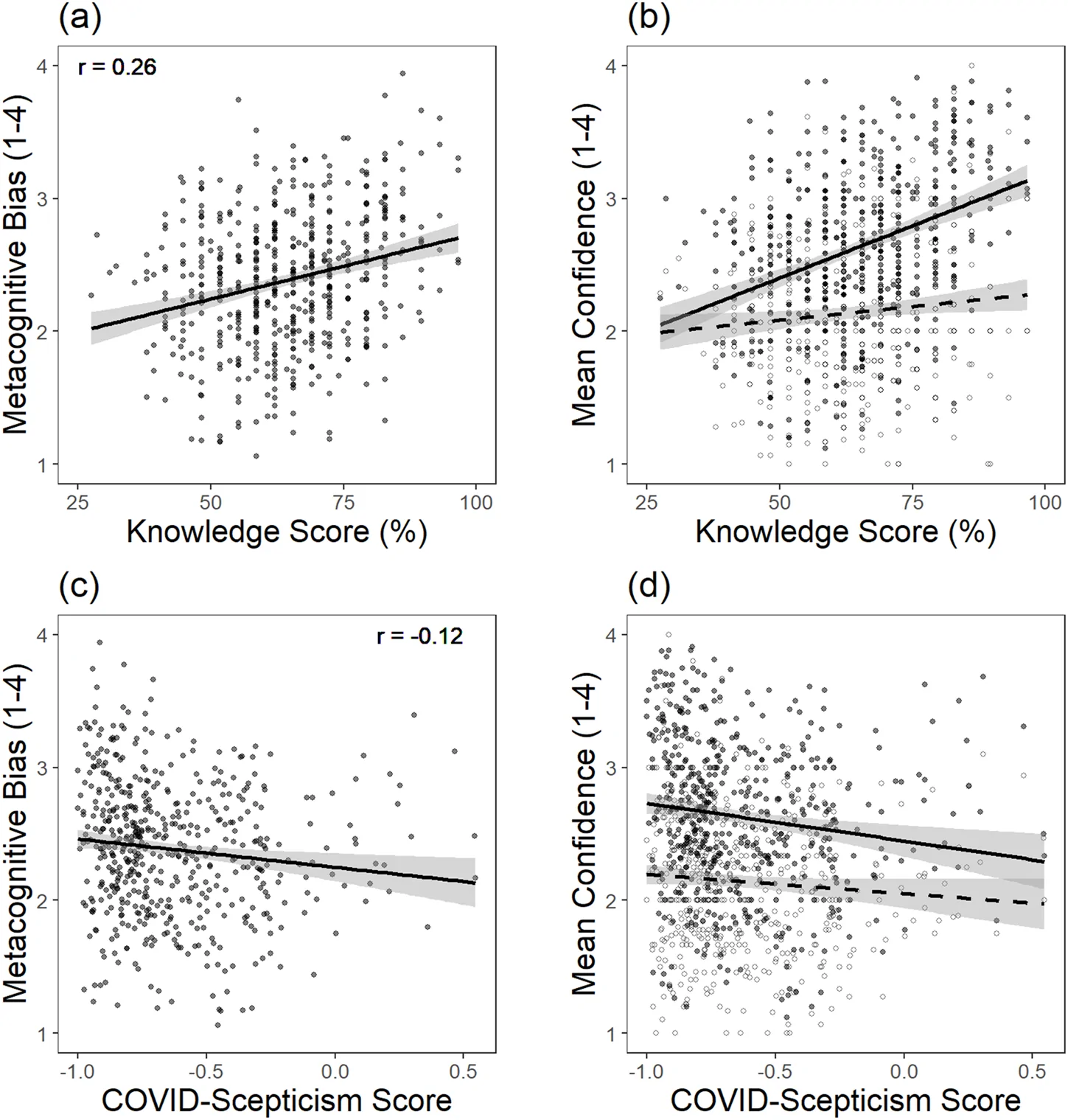
Panels (a) and (b) show relationships between domain-relevant knowledge score and metacognitive bias and between knowledge score and mean confidence rating for correct and incorrect answers (solid and dotted lines, respectively). Panels (c) and (d) show relationships between total Covid-scepticism score and the same summary measures of confidence. Shaded areas show 95% CIs around the line of best fit.

Figure 3b unpacks the confidence ratings according to whether the first-order response was correct or incorrect. All people were unconfident to roughly the same degree (less than 2.5) when answering problems incorrectly, but higher performers were more confident when answering correctly. Higher performers have a clear separation between their (average) confidence ratings for correct and incorrect answers, showing that they are sensitive to the quality of their first-order knowledge in a way that the poorer performers are not. Nonetheless, the poorer performers did seem to be globally sensitive to their poor level of knowledge: the confidence of the worst performers, for correct and incorrect answers alike, was approximately equivalent to the (low) confidence that the highest performers had in their incorrect answers.

Finally, we note that higher CS scores, being related to lower knowledge scores (figure 2d), inherit a negative relationship with confidence. As illustrated in figure 3c,d, higher CS tended to predict lower metacognitive bias (*r* = −0.12, *p* = 0.005), and less distinction between the (low) confidence expressed for correct and incorrect answers on the knowledge test. CS was, therefore, associated with lower confidence in the knowledge test, even when taking account of the objectively lower level of domain-relevant knowledge.

## 4. Discussion

The present study aimed at evaluating a widespread claim in public discourse that vaccine-scepticism can be explained by the DKE, whereby individuals with low knowledge are both ignorant and unaware of that ignorance. Combining a newly developed, psychometrically grounded CS scale with a domain-relevant knowledge test and item-level confidence ratings, the study was designed to avoid methodological pitfalls that have compromised prior work in this area (e.g. [9,46]). The results are inconsistent with the core claims of the DKE narrative as applied to vaccine-scepticism and vaccine hesitancy and instead paint a more nuanced picture of the relationships between scepticism, knowledge and confidence.

### 4.1. Vaccine-scepticism and domain-relevant knowledge

CS should not be viewed as a unitary construct, and the present findings should be interpreted in light of the specific form of knowledge that was assessed. Our knowledge test was designed to measure biological knowledge, historical knowledge concerning infectious disease and probabilistic reasoning relevant to understanding a viral pandemic. It was not intended as a measure of political, institutional or social knowledge, which are also relevant to forming attitudes to the pandemic. Consequently, the observed association between CS and lower knowledge should not be taken to demonstrate a general deficit in understanding. Rather, it indicates a modest reduction in knowledge within the particular scientific domains most directly relevant to evaluating claims about viral transmission, vaccination and disease risk.

In our data, CS attitudes were associated with lower levels of pandemic-relevant knowledge relevant to understanding infectious disease and public health information, predicting a 12.5 percentage-point drop in the knowledge test per unit increase on the CS scale (equivalent to a shift from complete CO to neutrality). Previous research into CS attitudes has usually focused on conspiracy theories and conspiracy-adjacent beliefs, such as that Covid-19 symptoms are caused by 5G electromagnetic radiation or that the virus itself is a hoax (e.g. [12,48–51]). It is well established that endorsement of one conspiracy theory is associated with endorsement of others [47,52,53], and Covid-related conspiracy beliefs appear to conform to this so-called monological pattern [54,55]. The validation against the GCB scale in the present data confirms that people who score high on the CS scale also present more conspiracy thinking and vaccine hesitancy. Although it is possible that different outcomes would be observed in samples with more extreme CS attitudes, we have no reason to believe that such extreme attitudes were under-represented in our sample relative to the UK population.

However, correlations between conspiracy beliefs do not by themselves prove a monological attitude (for a discussion see [56,57]). Indeed, all three latent subfactors, severity/policy scepticism, vaccine/conspiracy scepticism and health/mechanism scepticism, showed positive correlations with both external measures, as did the overall CS score (table 3). These findings suggest that CS should be seen as a broad attitudinal disposition rather than a narrow endorsement of fringe conspiracy theories alone. The associations with severity/policy and health/mechanism scepticism show that doubts about the proportionality of public health measures and about mainstream biomedical explanations also intersect with vaccine hesitancy. This broader framing is consistent with the view that vaccine-scepticism is emerging from a constellation of beliefs about risk, trust, authority and institutional credibility, rather than from conspiracy ideation in isolation [58].

Eleven of the 23 items in our CS scale were assigned to a factor relating to vaccine-scepticism and conspiracy thinking, and the vaccine/conspiracy sub-score showed the strongest (negative) relationship with the knowledge test, consistent with the idea that domain-relevant knowledge is relevant to vaccination attitudes (see table 4 and figure 2). These findings align with studies showing that scientific literacy, probabilistic reasoning and analytical thinking are positively associated with acceptance of public health guidance and vaccination (e.g. [10,12]). However, vaccine-scepticism accounted for a relatively modest proportion of variance in knowledge scores, underscoring that a sceptical attitude cannot be reduced to a mere lack of information. This is consistent with broader perspectives on vaccine hesitancy, which emphasize the roles of diverse factors, including trust, political identity, institutional confidence and social context alongside knowledge deficits [59,60].

Similar considerations hold for CS conceived more broadly, and it is notable that the severity/policy sub-scale was relatively weakly related to the knowledge score, perhaps because it can be rational for a person who knows the relevant science to nonetheless judge that the health risks of coronavirus do not justify official policies. Indeed, although severity/policy was (weakly) negatively associated with domain-relevant knowledge overall, this association became (weakly) *positive* when variance shared with the other CS sub-scores was accounted for (see §3.3). One possible interpretation is that, while the variance shared among the sub-scales reflects a general rejection of mainstream messaging, the *unique* (positive) relationship between severity/policy scepticism and knowledge may reflect more informed critical evaluation of policy decisions. Our data do not allow this interpretation to be tested, and the relationship is minor and somewhat peripheral to the main thrust of our findings.

Importantly, the present study avoided measurement circularity in assessing the relationship between attitudes and knowledge, by avoiding direct overlap between the items assessing these constructs, therefore avoiding the problem of labelling as ignorance any opposition to mainstream messages. As such, the observed associations are unlikely to be artefacts of tautological item construction, strengthening confidence that the relationship reflects genuine (albeit limited), covariance between scepticism and domain-relevant knowledge. However, the overall distribution of CS scores highlights an important contextual point (figure 1; supplementary material, figure S1.2). The sample was overwhelmingly CO, with a vanishingly small minority expressing strongly sceptical attitudes. CS in this study should be understood as a relative bias along a continuum, rather than as membership of a distinct or extreme subgroup. This framing is important when interpreting both knowledge and confidence effects and cautions against caricatures of CS as a distinct, radical group. It also dispels any impression that extreme CS attitudes are common in the population.

### 4.2. Knowledge, confidence and metacognitive sensitivity

A central strength of this study is in the use of item-level confidence ratings to probe metacognitive insight, rather than relying on discrepancy-based measures of global self-overestimation, which are prone to statistical artefacts [17–23]. By averaging confidence ratings across correct and incorrect trials, we computed metacognitive bias, which is unconfounded with first-order knowledge level [15]. This methodological choice allows for more defensible inferences about whether low performers are genuinely biased towards overconfidence.

Contrary to the popular DKE narrative, poorer performers on the knowledge test were not excessively confident. On the contrary, metacognitive bias increased with knowledge, such that higher performers showed a clear (and appropriate) bias to express higher confidence. Higher-performing participants also showed clearer differentiation between correct and incorrect answers, expressing greater confidence when correct and lower confidence when incorrect, consistent with the pattern that metacognitive sensitivity improves with expertise [22]. Lower performers expressed relatively low confidence across the board, including for their correct responses; their feelings of confidence were thus less predictive of whether their responses were correct or not, indicating a lack of metacognitive sensitivity. However, this lack of insight was not accompanied by overconfidence; rather, poorer performers were broadly aware that their knowledge was unreliable, expressing lower confidence overall. According to this analysis, the popular narrative associated with the DKE, of unwarranted confidence born of unrecognized incompetence, was not apparent. The results converge with prior demonstrations that apparent DKE patterns often disappear, or reverse, when confidence is measured appropriately, rather than inferred from discrepancy-based measures of global self-assessment (e.g. [22,23]).

### 4.3. Covid-scepticism is not associated with overconfidence

If CS were driven by a DKE-style deficit in metacognition (i.e. unawareness of one's own ignorance), then sceptical individuals should not only have less knowledge but also express inflated confidence in that knowledge. The data are inconsistent with this prediction, because CS was associated with lower, not higher, metacognitive bias (figure 3c,d). Far from being overconfident, more sceptical individuals appeared more uncertain, expressing generally lower confidence, albeit this pattern was relatively weak.

This finding is relevant given the prevalence of recurrent claims that vaccine-hesitant or vaccine-sceptic individuals ‘think they know more than experts’. Such claims may be rhetorically powerful but are empirically weak, beyond the self-evident fact that the person expresses a belief that diverges from the expert consensus. The present results suggest that scepticism is better characterized by disengagement from, or distrust of, mainstream sources of information (and/or misplaced trust in other, self-proclaimed ‘experts’), rather than by a conceited self-assurance in one's own knowledge. This interpretation is a plausible inference from the confidence and knowledge patterns observed, consistent with a substantial body of work emphasizing mistrust as central to sceptical attitudes [61–64]. However, it is not a claim directly demonstrated by the present study, which did not independently measure trust, institutional credibility or information-seeking behaviour. Direct evidence for this mechanism would require designs that assess these factors explicitly. A possible alternative account relies on discussions of medical healthism [65], which have argued that vaccine-related attitudes may be embedded within moralized understandings of health, whereby individuals place particular emphasis on personal responsibility, self-management and bodily autonomy [66] also related to Covid [67]. From this perspective, vaccine-scepticism need not arise from a lack of insight into one's state of knowledge but may instead reflect coherent, even if ill-advised, beliefs regarding health and risk. Our findings do not address such accounts directly, but the evidence that higher levels of scepticism were not associated with overconfidence does suggest the need for explanatory accounts beyond metacognitive deficit models.

### 4.4. Implications for the Dunning–Kruger effect in applied contexts

The tendency to explain vaccine hesitancy and vaccine-scepticism through the lens of the DKE has been rhetorically powerful but is scientifically fragile. By portraying sceptical individuals as ignorant and overconfident, the narrative risks fostering dismissive attitudes, reinforcing social divisions and undermining constructive dialogue. The present findings suggest that such portrayals are not empirically justified. While sceptical individuals may indeed possess less accurate knowledge on average, they do not appear to be blind to this fact and even express slightly lower confidence in their knowledge of relevant domains.

This distinction matters for public health communication. If scepticism were driven by overconfidence, interventions might focus on fostering humility or highlighting one's own ignorance. However, if scepticism is associated instead with lower confidence and uncertainty, then effective interventions may need to be better-designed to focus on trust, clarity and the perceived credibility of information sources. Public commentaries, policies and interventions based on caricatures of sceptics as arrogantly ignorant risk being not only inaccurate but counterproductive, potentially exacerbating mistrust and social division.

The findings also contribute to ongoing debates about the scope and interpretation of the DKE. The much-discussed pattern, whereby low performers appear overconfident (and high performers underconfident), has been shown to arise from statistical artefacts when discrepancy scores are used [21–23,31]. Avoiding these methods, the present study shows that, in a socially and politically relevant domain, the DKE construct provides little explanatory value. This does not imply that metacognitive limitations are irrelevant to public understanding of science, only that ignorance and overconfidence should not be conflated. People may lack knowledge, and metacognitive insight about that knowledge, while simultaneously being more broadly aware that their knowledge is limited.

### 4.5. Limitations

Some limitations should be acknowledged. First, although the sample was broadly representative of the UK population, it contained relatively few participants at the extreme sceptical end of the scale. As such, the conclusions apply most directly to variations in scepticism within a largely CO population and cannot necessarily be generalized to the (hypothetical) case of an extreme CS, albeit the data also imply that such individuals may be vanishingly rare in the population. Second, our measure of pandemic-relevant knowledge was biased towards scientific and mathematical knowledge; we did not assess social, economic or political dimensions that would also be relevant to pandemic-relevant decision-making. Finally, the cross-sectional nature of the data limits causal inference. It remains to be determined whether lower knowledge contributes to scepticism, whether scepticism leads to disengagement from information acquisition, or whether both are shaped by third variables such as political ideology, institutional trust or prior experience with health systems. All three pathways are plausible, and they are not mutually exclusive. Longitudinal and experimental approaches will be needed to disentangle these directions of effect.

## 5. Conclusions

The present study challenges the idea that CS, and by extrapolation vaccine-scepticism, is driven by a lack of knowledge associated with overconfidence, as narratives based on the DKE assume. CS is indeed associated with somewhat lower levels of domain-relevant knowledge, but sceptical individuals are not overconfident about that knowledge. Instead, they tend to express lower confidence overall, suggesting appropriate global self-awareness rather than ignorance of their epistemic limitations. These findings caution against simplistic psychological explanations of public health scepticism and highlight the importance of rigorous measurement and theory when applying cognitive constructs to real-world social issues.

## Supplementary Material



## Data Availability

The full raw and processed data, with analysis code, are archived at [68]. Supplementary material is available online [69].
