# Supplementary material for: The Dunning–Kruger effect does not account for Covid-scepticism or vaccine hesitancy
Source: R Soc Open Sci. 2026 Oct 7;13(10):260147. doi: 10.1098/rsos.260147 (PMC13641890; doi:10.1098/rsos.260147)
Supplement: Supplementary file 1 [file rsos.260147.zip › rsos.260147/rsos260147_si_001.pdf]

SUPPLEMENTARY MATERIALS

The Dunning-Kruger effect does not account for Covid-Scepticism or vaccine-hesitancy

Robert D. McIntosh, Justin Claydon, Tom Booth & Sergio Della Sala

The full raw and processed data, with analysis code, are archived at https://osf.io/bjhwz.

S1. Psychometric development of a Covid-Scepticism scale

S1.1. Item generation, and face-validity

To develop a novel, valid and reliable Covid-Scepticism scale, we operationalised Covid-Scepticism as the strength of endorsement of Covid-Sceptic (CS) statements, and rejection of Covid-Orthodox (CO) statements, where CO statements reflect mainstream narratives surrounding the pandemic, and CS statements reject those narratives, or reflect alternative narratives. In generating candidate items for this scale, we aimed to include a range of themes/dimensions of Covid-Scepticism, without attempting to be comprehensive. Item generation was led by author JC, based on information from mainstream media, and scrutiny of online discussions and social media. In particular, the content was informed by a website established during the pandemic: 'Anti-virus: The Covid-19 FAQ'.[1] The 'Anti-virus' website (now discontinued) had been created by a group of academics, journalists and volunteers to identify and counteract misinformation about Covid-19. The items generated by JC were subjected to two rounds of review for face-validity, and refinement of wording by a panel of the three authors (JC, SDS, RDM) and Dr. Stuart Ritchie, one of the chief architects of the Anti-virus website, then a lecturer at Kings College London. These discussions took place via email, shared collaborative documents, and live online meetings, and they resulted in a refined list of 72 candidate statements for the scale.

The 72 statements comprised 36 conceptual items, each composed of two opposed statements, one expressing a CS attitude, and the other expressing a CO attitude. The 36 items were distributed evenly across six broad themes: *Conspiracy*, *Mechanism*, *Policy*, *Severity*, *Treatment*, and *Vaccine*. These themes were used as guidance to ensure that our scale covered a range of different aspects of Covid-Scepticism; but they were not rigidly distinct, and they did not represent predictions for components (latent factors) of Covid-Scepticism. Table S1.1 shows the complete set of 72 statements for the 36 candidate items.

---

[1] The website, which was taken down after the pandemic, had the address http://www.Covidfaq.co/

Table S1.1: *The complete set of 72 statements for 36 candidate conceptual items, each composed of two opposed statements, one Covid-Sceptic (CS) and the other Covid-Orthodox (CO). Six a priori themes were used for guidance in generation of items. Dark grey shading indicates two items (n. 21 and 23) that were excluded from analysis for producing inconsistent responses; light grey shading indicates ten items (n.1,5,10, 12, 14, 15, 24, 26, 27, 33) that were excluded after the first round of EFA; very dark indicates one item (n.22) that was excluded after the second round of EFA. The 23 unshaded items constitute the final item set selected for the main study.*

Theme	Item	Polarity	Statement
Conspiracy	1	CS	The need for lockdowns is a Chinese Communist Party fraud, to slow down rival economies.
Conspiracy	1	CO	The need for lockdowns is not a Chinese Communist Party fraud, to slow down rival economies.
Conspiracy	2	CS	Covid-19 vaccines contain technology that allows people to be tracked.
Conspiracy	2	CO	Covid-19 vaccines do not contain technology that allows people to be tracked.
Conspiracy	3	CS	Patents for Covid-19 vaccines were filed before the outbreak of the pandemic.
Conspiracy	3	CO	No patents for Covid-19 vaccines were filed before the outbreak of the pandemic.
Conspiracy	4	CS	The Bill and Melinda Gates Foundation owned a patent for a Covid-19 vaccination prior to the outbreak of Covid-19.
Conspiracy	4	CO	The Bill and Melinda Gates Foundation did not own a patent for a Covid-19 vaccination prior to the outbreak of Covid-19.
Conspiracy	5	CS	Covid-19 is caused by 5G electromagnetic radiation.
Conspiracy	5	CO	Covid-19 is not caused by 5G electromagnetic radiation.
Conspiracy	6	CS	Covid-19 vaccines can alter a person's DNA.
Conspiracy	6	CO	Covid-19 vaccines cannot alter a person's DNA.
Mechanism	7	CS	Covid-19 cannot be transmitted by asymptomatic people.
Mechanism	7	CO	Covid-19 can be transmitted by asymptomatic people.
Mechanism	8	CS	Covid-19 is not spread by liquid droplets in the air (aerosols).
Mechanism	8	CO	Covid-19 is spread by liquid droplets in the air (aerosols).
Mechanism	9	CS	Long-term after effects of Covid-19 do not exist (“long Covid”).
Mechanism	9	CO	Long-term after effects of Covid-19 exist (“long Covid”).
Mechanism	10	CS	Allowing Covid-19 to spread does not encourage the evolution of new variants.
Mechanism	10	CO	Allowing Covid-19 to spread encourages the evolution of new variants.
Mechanism	11	CS	Covid-19 does not spread more easily indoors.
Mechanism	11	CO	Covid-19 spreads more easily indoors.
Mechanism	12	CS	Close contact between individuals does not increase the chance of spreading of Covid-19.
Mechanism	12	CO	Close contact between individuals increases the chance of spreading of Covid-19.
Policy	13	CS	Quarantine is an unnecessary response to Covid-19.
Policy	13	CO	Quarantine is a necessary response to Covid-19.
Policy	14	CS	Lockdowns have only been introduced when Covid-19 cases were in decline.
Policy	14	CO	Lockdowns have only been introduced when Covid-19 cases were rising.

Policy	15	CS	A natural herd immunity approach to Covid-19 (i.e., without lockdowns/vaccines) is a good idea.
Policy	15	CO	A natural herd immunity approach to Covid-19 (i.e., without lockdowns/vaccines) is not a good idea.
Policy	16	CS	Lockdowns do not reduce the spread of Covid-19.
Policy	16	CO	Lockdowns reduce the spread of Covid-19.
Policy	17	CS	Measures to reduce the spread of Covid-19 are not needed to protect the health service.
Policy	17	CO	Measures to reduce the spread of Covid-19 are needed to protect the health service.
Policy	18	CS	Governments should not be able to impose restrictions on personal freedom in order to combat Covid-19.
Policy	18	CO	Governments should be able to impose restrictions on personal freedom in order to combat Covid-19.
Severity	19	CS	The infection fatality ratio of Covid-19 is low enough that we should not be concerned.
Severity	19	CO	The infection fatality ratio of Covid-19 is high enough that we should be concerned.
Severity	20	CS	Covid-19 is not serious enough to justify the measures being taken across the world.
Severity	20	CO	Covid-19 is serious enough to justify the measures being taken across the world.
Severity	21	CS	Covid-19 is only dangerous to you if you're elderly or have certain pre-existing health conditions.
Severity	21	CO	Covid-19 is not only dangerous if you're elderly or have certain pre-existing health conditions.
Severity	22	CS	Your chances of dying if you catch Covid-19 are not higher than if you catch seasonal flu.
Severity	22	CO	Your chances of dying if you catch Covid-19 are higher than if you catch seasonal flu.
Severity	23	CS	Covid-19 is not causing excess deaths, relative to people that would have died in the same period anyway.
Severity	23	CO	Covid-19 is causing excess deaths, relative to people that would have died in the same period anyway.
Severity	24	CS	Covid-19 is no more contagious than the flu.
Severity	24	CO	Covid-19 is more contagious than the flu.
Treatment	25	CS	Cheap medical treatments like Vitamin D and Ivermectin would prevent or treat Covid-19 effectively.
Treatment	25	CO	Cheap medical treatments like Vitamin D and Ivermectin would not prevent or treat Covid-19 effectively.
Treatment	26	CS	Wearing masks causes health problems and infections.
Treatment	26	CO	Wearing masks does not cause health problems and infections.
Treatment	27	CS	Masks reduce the wearer's oxygen supply.
Treatment	27	CO	Masks do not reduce the wearer's oxygen supply.
Treatment	28	CS	Masks do not reduce the spread of Covid-19.
Treatment	28	CO	Masks reduce the spread of Covid-19.
Treatment	29	CS	Social distancing does not reduce the spread of Covid-19.
Treatment	29	CO	Social distancing reduces the spread of Covid-19.
Treatment	30	CS	Vaccines cannot reduce the symptoms and transmission of Covid-19.
Treatment	30	CO	Vaccines can reduce the symptoms and transmission of Covid-19.
Vaccine	31	CS	Vaccines for Covid-19 have caused an increase in care home deaths
Vaccine	31	CO	Vaccines for Covid-19 have caused a decrease in care home deaths.
Vaccine	32	CS	Covid-19 vaccines are unsafe.

Vaccine	32	CO	Covid-19 vaccines are safe.
Vaccine	33	CS	People receiving Covid-19 vaccines do not give their informed consent.
Vaccine	33	CO	People receiving Covid-19 vaccines give their informed consent.
Vaccine	34	CS	Covid-19 vaccines are ineffective.
Vaccine	34	CO	Covid-19 vaccines are effective.
Vaccine	35	CS	The fact that there is ongoing monitoring of Covid-19 vaccines indicates they are unsafe.
Vaccine	35	CO	The fact that there is ongoing monitoring of Covid-19 vaccines DOES NOT indicate they are unsafe.
Vaccine	36	CS	Clinical trials of the approved Covid-19 vaccines are insufficiently conclusive.
Vaccine	36	CO	Clinical trials of the approved Covid-19 vaccines are sufficiently conclusive.

S1.2. Participants

A total of 357 adult participants were recruited from an online participant pool (Prolific Academic)[2], between 5 July and 6 August, 2021. The sample was restricted to participants living in the UK or the US, but no other restrictions were applied. The sample size was determined as the largest sample size that resources available at the time would allow. The survey took a median time of 10 m 51 s to complete, and participants were compensated at a standard rate for completion. The study was approved by the PPLS Research Ethics Committee at the University of Edinburgh.

The final sample (after all exclusions) had 307 participants, the demographic details for whom were provided by Prolific. Participants had the option to withhold demographic data, and some demographic data had expired (were no longer available) at the time that they were downloaded; the n values below indicate the size of the sub-sample that each summary is based on. There were 73% women and 27% men (n = 287), with a mean age of 27.8, SD 8.9 (n = 305); 56% were resident in the US and 44% in the UK (n = 305); and English was the first language for 86% participants (n = 283). All participants answered the question about Covid vaccination status: 269 (87.6%) reported having received a vaccine, 21 (6.8 %) reported that they would not accept if offered, and 17 (5.5%) were unsure.

S1.3. Materials and procedure

The online survey, created and administered using Qualtrics,[3] included the following components, in order: Covid-Scepticism scale; General Conspiracy Beliefs scale; additional

[2] http://prolific.co/
[3] http://www.qualtrics.com

questions. The procedure for these parts was essentially identical to that reported for the main experiment in the main paper, with the exception that the Covid-Scepticism scale encompassed 36 candidate items (72 statements) (Table S1.1), rather than the 23 items (46 statements) selected from these candidates for the final scale (Table 1, main paper).

S1.4. Data processing and scoring

Scoring

The scoring of all parts of the survey followed an identical procedure to that reported for the main experiment.

Participant exclusions

From the total sample of 357 participants, 19 were excluded because they did not complete the entire survey. Three further participants had not responded to either statement for one of the items on the Covid-Scepticism scale, so these cases were also considered incomplete and excluded. The remaining sample of 335 participants was entered into the analysis.

Item exclusions

For the remaining 335 participants, we examined the rates of discrepant responses for each of the 36 items, where a discrepant response was defined as a difference greater than one (i.e., more than two notches on the Likert-scale) between responses to the CS and CO statements for that item. Based on a visual inspection of the distribution of differences, we excluded any item with a discrepancy rate >10%, as this item evidently did not elicit equal and opposite responding to the CS and CO versions of the statement. This criterion led to the exclusion of two items (shaded red in Table S1.1), leaving 34 items in the analysis.

S1.5. Exploratory Factor Analysis, and further exclusions

We conducted Exploratory Factor Analysis (EFA) to investigate the underlying structure of Covid-Scepticism attitudes as measured by our scale. All EFA analyses were estimated based on maximum likelihood extraction, using Oblimin (oblique) rotation, as we expected the factors to be correlated. The Kaiser-Meyer-Olkin (KMO) test confirmed the factorability of the data, yielding an overall KMO of 0.95, and Bartlett's test of sphericity indicated that a factor analysis may be useful ($\chi^2(561) = 6016, p < .00001$).

At the first round of EFA (34 items), the minimum average partial value had its minimum at three factors, whilst parallel analysis based on component extraction suggested three factors

(or four factors based on a factor extraction method). Visual inspection of the scree plot suggested to retain three factors. Figure S1.1 shows a diagnostic plot for each method. On the balance of these results, and considering theoretical coherence of the solution, we chose to retain three factors. These factors accounted for 48% of the variance in item scores at the first round, and the model fit was good (RMSEA index 0.058; Tucker Lewis Index 0.89).

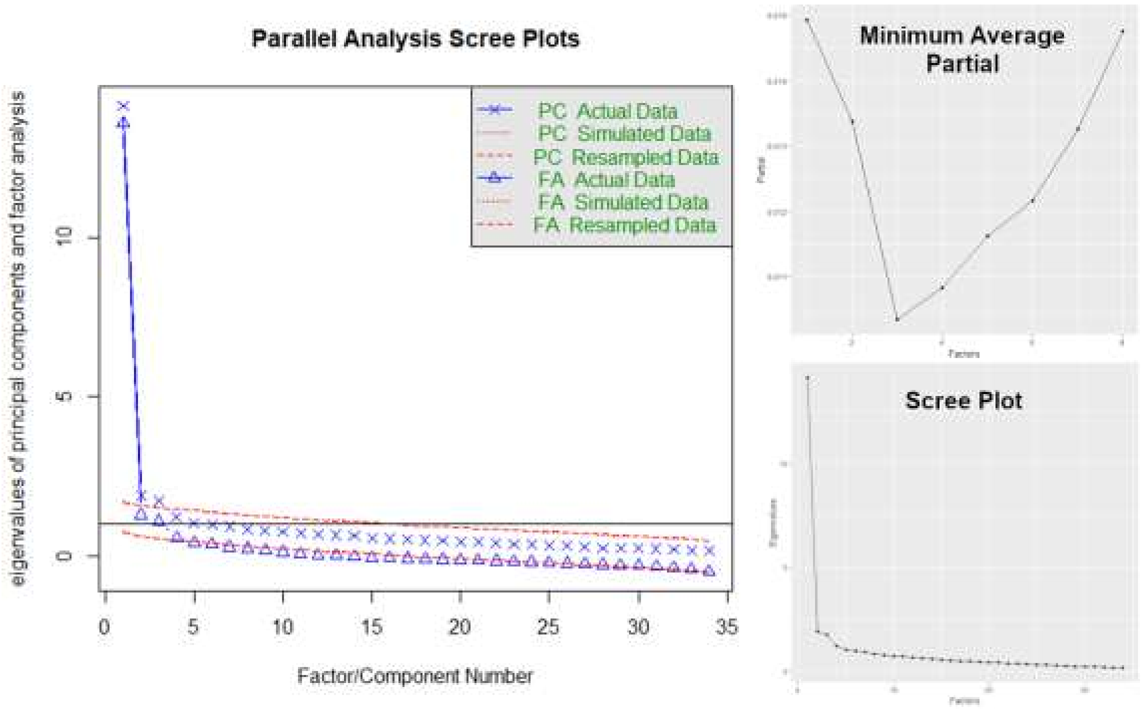

Figure S1.1. *Diagnostic plots for the optimal number of factors to extract.*

The filtered dataset of 335 complete cases for 34 items was entered into a first round of EFA, and the set of items was iteratively reduced until an optimal factor structure emerged. At each iteration, the dataset was restricted to the remaining items of interest, and filtered by excluding any participant with more than 10% discrepant responses across those items. The refinement of items was guided at each iteration by the following three criteria. First, all retained factors should have at least three primary loading items. Second, items with complexity values greater than 50% of the maximum for a given solution (i.e., the number of factors) are deemed to load insufficiently cleanly onto a single factor, and excluded. Third, all items should have at least one primary loading greater than 0.30.

In the first round, there were 34 items, ten of which (shaded orange in Table S1.1) loaded insufficiently cleanly onto one factor (complexity > 1.5), and were excluded for the second

round. In the second round, there were 24 items, one of which (shaded yellow in Table S1.1) loaded insufficiently cleanly onto one factor (complexity > 1.5), and was excluded for the third round. The third round was based on 23 items (unshaded in Table S1.1) and 307 complete (non-discrepant) cases, and yielded the optimal factor model described below.

The data for the final EFA had an overall KMO of 0.94, and Bartlett's test of sphericity was passed ($\chi^2(253) = 4038, p < .00001$). The three factors accounted for 52% of the variance, and the model fit was good (RMSEA index 0.062; Tucker Lewis Index 0.92). Factor 1 and 2 were strongly correlated (r = .70), whilst Factor 3 was more moderately correlated with both Factor 1 (r = .47) and Factor 2 (r = .40). The factor loadings for each of the 23 included items are presented in Table S1.2 with each item represented by its CS statement (see Table S1.1 for the paired CO statements).

S1.6. Factor interpretation

The initial themes used for item generation are listed in Table S1.2 and are related strongly to the objective grouping of items by factor, with *Policy* and *Severity* items clustering in Factor 1, *Conspiracy* and *Vaccine* items clustering in Factor 2, and *Mechanism* items allocated to the minor Factor 3. The exception to these neat groupings by initial theme was for *Treatment* theme items, which were split between Factor 1 and Factor 3. However, the allocation of these items makes sense in terms of their specific content: the items allocated to Factor 1 (Items 28 and 29) concerned the effects of masks and social distancing, which are also aspects of policy; and the items allocated to Factor 2 (Items 30 and 25) concerned scepticism about Covid vaccines, and belief in cheap treatments such as Vitamin D and Ivermectin, which can be considered conspiracy-adjacent beliefs as they often involve the idea that this information is being suppressed by vested interests.

Therefore, Factor 1 we labelled 'Severity/Policy Scepticism', as the items grouped around the idea that government policies to reduce the spread of the virus are disproportionate to the severity of the risk it poses. Factor 2 we labelled 'Vaccine/Conspiracy Scepticism', as the items grouped around the effectiveness and safety of Covid vaccines, and pandemic-related conspiracy theories. Factor 3 we labelled 'Health/Mechanism Scepticism', as the items concerned mainstream messages about how the virus is spread and the health risks it poses.

Table S1.2: *Exploratory Factor Analysis Table for the final reduced model. Extraction method minimum residual. Rotation method Oblimin, Based on clustering of a priori themes, and consideration of specific item content, Factors 1-3 were given the following labels respectively: Severity/Policy Scepticism; Vaccine/Conspiracy Scepticism; Health/Mechanism Scepticism.*

**Item**	**Theme**	**Statement (CS version)**	**Factor 1**	**Factor 2**	**Factor 3**
20	Severity	Covid-19 IS NOT serious enough to justify the measures being taken across the world.	**0.89**	0.02	-0.06
16	Policy	Lockdowns DO NOT reduce the spread of Covid-19.	**0.84**	-0.08	0.11
13	Policy	Quarantine is an unnecessary response to Covid-19.	**0.83**	0.02	-0.10
19	Severity	The infection fatality ratio of Covid-19 is low enough that we SHOULD NOT be concerned.	**0.73**	0.08	-0.06
28	Treatment	Masks DO NOT reduce the spread of Covid-19.	**0.72**	0.04	0.08
17	Policy	Measures to reduce the spread of Covid-19 ARE NOT needed to protect the health service.	**0.70**	0.00	0.19
18	Policy	Governments SHOULD NOT be able to impose restrictions on personal freedom in order to combat Covid-19.	**0.61**	0.21	-0.09
29	Treatment	Social distancing DOES NOT reduce the spread of Covid-19.	**0.59**	0.12	0.19
32	Vaccine	Covid-19 vaccines are unsafe.	0.08	**0.79**	-0.06
4	Conspiracy	The Gates Foundation owned a patent for a Covid-19 vaccination prior to the outbreak of Covid-19.	-0.14	**0.72**	-0.06
35	Vaccine	The fact that there is ongoing monitoring of Covid-19 vaccines indicates they are unsafe.	-0.03	**0.70**	0.17
34	Vaccine	Covid-19 vaccines are ineffective.	0.19	**0.66**	0.05
3	Conspiracy	Patents for Covid-19 vaccines were filed before the outbreak of the pandemic.	-0.04	**0.64**	-0.14
30	Treatment	Vaccines CANNOT reduce the symptoms and transmission of Covid-19.	0.10	**0.63**	0.05
36	Vaccine	Clinical trials of the approved Covid-19 vaccines are insufficiently conclusive.	0.08	**0.62**	0.07
2	Conspiracy	Covid-19 vaccines contain technology that allows people to be tracked.	-0.02	**0.58**	0.17
6	Conspiracy	Covid-19 vaccines can alter a person's DNA.	0.08	**0.52**	0.02
31	Vaccine	Vaccines for Covid-19 have caused an increase in care home deaths	0.01	**0.47**	0.09
25	Treatment	Cheap medical treatments like Vitamin D and Ivermectin would prevent or treat Covid-19 effectively.	0.12	**0.47**	-0.04
9	Mechanism	Long-term after effects of Covid-19 DO NOT exist (“long Covid”).	0.01	0.03	**0.72**
7	Mechanism	Covid-19 CANNOT be transmitted by asymptomatic people.	0.20	0.00	**0.63**
11	Mechanism	Covid-19 DOES NOT spread more easily indoors.	0.02	0.13	**0.59**
8	Mechanism	Covid-19 IS NOT spread by liquid droplets in the air (aerosols).	-0.09	0.13	**0.52**

S1.7. Covid-Scepticism score, factor sub-scores, and external validity

For each participant, the three factor sub-scores were calculated as the unweighted mean of the items in each factor, and an overall Covid-Scepticism Score was calculated as the mean of three factor sub-scores. Figure S1.2 shows that each of the factor scores had a positively-skewed distribution, with a compression at the left, because many people scored at or close to a minimum level of Covid-Scepticism (i.e. maximum Covid-Orthodoxy). As in the main study (see Figure 1, main paper), we did not see any participants with strongly Covid-Sceptic views; rather, Covid-Scepticism was mainly a matter of weaker endorsement of orthodoxies. The factor sub-scores intercorrelated substantially, with r between .46 and .70 (Spearman correlations were similar in all cases).

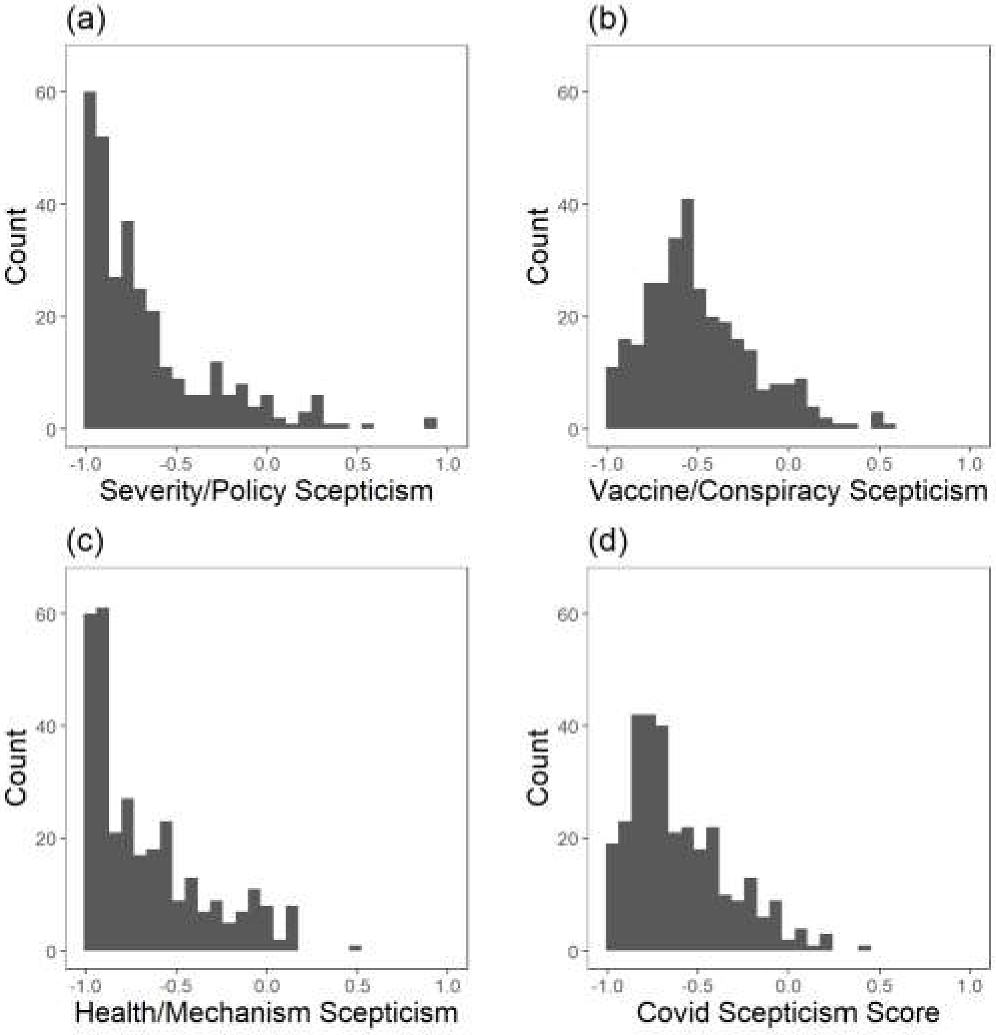

Figure S1.2: *Distribution of scores for the Covid-Scepticism latent sub-factors (panels a-c), and for the total Covid-Scepticism Score (panel d), in preliminary (Exploratory Factor Analysis) study.*

We were able to check the Covid-Scepticism scale for external validity with respect to conspiracy thinking (as measured by the General Conspiracy Beliefs scale), and Covid-vaccine hesitancy (as measured by the further questions about vaccinations). These measures are most directly relevant to the Vaccine/Conspiracy factor; and, unsurprisingly, the Vaccine/Conspiracy sub-score correlated most strongly with these items, although positive correlations (with confidence intervals not including zero), were observed for all factors and for the overall Covid-Scepticism score (Table S1.3).

Table S1.3. *Correlations (and 95% CIs) of factor sub-scores and overall Covid-Scepticism Score with General Conspiracy Beliefs and Covid Vaccine Hesitancy. The latter is a binary classification, so the correlations in the right column are point-biserial.*

	**General Conspiracy Beliefs**	**Covid Vaccine Hesitancy**
**Severity/Policy Scepticism**	.35 [.26, .44]	.49 [.35, .60]
**Vaccine/Conspiracy Scepticism**	.55 [.47, .62]	.61 [.51, 0.68]
**Health/Mechanism Scepticism**	.32 [.21, .42]	.19 [.07, .31]
**Covid-Scepticism Score**	.48 [.38, .55]	.51 [.40, .61]

S1.8. Confirmatory Factor Analysis

The reduced, 23 item Covid-Scepticism scale was then used for the main study, for which 537 new valid datasets were available after exclusions (see Methods, in main paper). This allowed us to test the structure of the scale, by a Confirmatory Factor Analysis, which was conducted in lavaan version 0.6-9 (Rosseel, 2012). In accordance with the outcome of the EFA (Table S1.2), the model was specified to have three factors; Severity/Policy Scepticism comprising 8 items, Vaccine/Conspiracy Scepticism comprising 11 items, and Health/Mechanism Scepticism comprising 4 items. Latent factors were identified by fixing the variance of the latent factor to 1.0. All items were loaded on their primary factor, with no *a priori* cross-loadings included in the model. The model was estimated using maximum likelihood. Model fit was evaluated based on a range of model fit indices, and the typically accepted cut-offs for each index. We considered values < 0.08 for the Root Mean Square Error of Approximation (RMSEA), and of < 0.06 for the Standardized Root Mean Residual

(SRMR), as being indicative of a well-fitting model. We considered values of > 0.95 for the Confirmatory Fix Index (CFI) and the Tucker-Lewis Index (TLI), to indicate excellent model fit, and values > .90 to indicate acceptable model fit. Where evidence was present of model misfit, we explored the model modification indices (MI) and expected parameter change (EPC) to identify the source of misfit. Where modifications were deemed plausible, revised models were estimated including these.

Table S1.4. *Model fit indices for the Confirmatory Factor Analysis models of the Covid-Scepticism scale.*

Model	χ^2	Df	p-value	CFI	TLI	RMSEA	SRMR
Original	992.34	227	< .001	0.89	0.88	0.079	0.052
Modified	711.79	224	< .001	0.93	0.92	0.064	0.047

Model fit statistics for the original and revised models are shown in Table S1.4. The original model showed good fit according to the SRMR and RMSEA, but was below the acceptable levels for the CFI and TLI, so we explored the modification indices to identify sources of misfit. Three large conceptually plausible modification indices were identified. In each case, these were residual correlations between pairs of items, indicating that after controlling for the shared variance due to the latent factor, items continued to be correlated. These item pairs were; items 2 and 3 (MI = 146.068) which both referenced vaccine patents; items 13 and 14 (MI = 70.541) which were the only remaining items concerning Covid severity; and items 16 and 17 (MI = 45.133) which both concerned preventative measures. [Note that item number here refers to those as listed in the final 23-item scale (see Table 1, main paper), not the original item numbers for the exploratory 36-item scale (Table S1.2).] On inclusion of these three parameters, model fit reached acceptable levels for all indices.

Model parameter estimates for the modified Confirmatory Factor Analysis model are shown in Table S1.5. All factor loadings were large (> 0.47) for all items. As is typical when moving from Exploratory to Confirmatory Factor Analysis, the factor correlations (shown at the bottom of the table) are notably higher due to the restriction of all cross-loadings to zero.

Table S1.5. *Standardized model parameters for the modified Confirmatory Factor Analysis model of the Covid-Scepticism scale. Residual correlations were: 0.52 for items 2 and 3; 0.377 for items 13 and 14; 0.32 for items 16 and 17. Item here refers to the item number as listed in the final scale (see Table 1, main paper), not the original item numbers (Table S1.2).*

Item	Severity/Policy Scepticism	Vaccine/Conspiracy Scepticism	Health/Mechanism Scepticism
IT09	0.836		
IT10	0.832		
IT11	0.836		
IT12	0.791		
IT13	0.700		
IT14	0.848		
IT16	0.763		
IT17	0.802		
IT01		0.601	
IT02		0.475	
IT03		0.487	
IT04		0.592	
IT15		0.511	
IT18		0.696	
IT19		0.653	
IT20		0.844	
IT21		0.848	
IT22		0.719	
IT23		0.794	
IT05			0.643
IT06			0.472
IT07			0.576
IT08			0.634
	1.00		
	0.697	1.00	
	0.735	0.721	1.00

S2. Psychometric development of a pandemic-relevant Knowledge Test

S2.1. Item generation

To create a test of pandemic-relevant knowledge, 57 True/False items. including biological and general knowledge relating to viruses, and questions assessing basic mathematical and probabilistic reasoning, were proposed by the researchers. These were refined via two rounds of small-scale piloting with friends and colleagues, for clarity of wording and general difficulty. This process yielded a set of 48 candidate items, shown in Table S2.1, which were entered into the more formal phase of piloting described below.

Table S2.1. *Forty-eight candidate knowledge items used in formal piloting. Shading indicates 18 items excluded from the final question set used in the main study.*

Item	Question	Answer (T/F)
Q01	The reproduction number (R) of an infectious disease can change	T
Q02	The word "vaccine" comes from a Latin word relating to cows	T
Q03	Viruses do not contain their own genetic material.	F
Q04	A mutation is a change in DNA	T
Q05	Malaria is caused by a virus.	F
Q06	SARS is caused by a virus	T
Q07	RNA stands for ribonucleic acid.	T
Q08	The largest known virus is 1 mm in diameter	F
Q09	There is a vaccine for Yellow Fever.	T
Q10	The Bubonic plague (A.K.A. "Black Death") is caused by a bacterium	T
Q11	A London doctor used a dot map of Diptheria, pioneering the modern science of epidemiology.	F
Q12	Viruses can reproduce on unattended surfaces	F
Q13	A disease vector is any agent which carries and transmits an infectious pathogen into another living organism.	T
Q14	Zoonotic diseases are diseases that have jumped from an animal to a human.	T
Q15	The incubation period is the time between symptom onset and recovery	F
Q16	The first viruses came from outer space	F
Q17	Using microscopes, viruses were discovered before bacteria	F
Q18	Bacteria and viruses are responsible for all infectious diseases	F
Q19	People can transmit infections during the prodromal stage	T
Q20	World War 1 had a bigger death toll than the 1918 Influenza Pandemic (a.k.a the Spanish Flu)	F

Q21	Antibiotics can kill viruses	F
Q22	Antiseptics can kill viruses	T
Q23	White blood cells are part of the immune system	T
Q24	Red blood cells are part of the immune system	F
Q25	Vaccines stimulate the body to produce more red blood cells.	F
Q26	Viruses can only infect humans and other animals	F
Q27	When infected, a host cell makes copies of a virus.	T
Q28	Viruses can only reproduce inside cells	T
Q29	One grain of rice, doubled every day for thirty days, will grow around ten thousand grains of rice	F
Q30	Zika is a virus spread by mosquitos	T
Q31	Fossil evidence shows that viruses have existed for longer than humans	F
Q32	Hepatitis is caused by viruses	T
Q33	The first outbreak of Ebola was in Brazil	T
Q34	Not all viruses that infect humans are harmful	T
Q35	The Human Papillomavirus (HPV) is sexually transmitted	T
Q36	Logistic growth describes an S-shaped growing curve	T
Q37	The infection rate is the total number of cases of a disease in a population	F
Q38	If a diagnostic test is 90% accurate, then there is a 9 in 10 chance you have the disease if you test positive	F
Q39	In a group of 100 people, it is almost certain that two people will share a birthday.	T
Q40	If I toss a coin and it lands on tails four times in a row, there is an 80% chance that it will land on heads on the next throw	F
Q41	If 60% of people living in City C get the Virus V, then living in City C is a risk factor for Virus V infection.	F
Q42	If a lottery draw has 10 prize tickets and 40 blanks, then the probability of getting a prize with one ticket is 1/5	T
Q43	If most children in bicycle accidents are boys, then a boy on a bicycle is at higher risk than a girl on a bicycle	F
Q44	Exponential growth means that the doubling time is increasing rapidly	F
Q45	The inverse of an exponential function is a logarithmic function	T
Q46	Viruses reproduce by splitting to produce two identical copies	F
Q47	Vaccines work on homeopathic principles	F
Q48	If you roll a six-sided dice, the chance of rolling an even number is 1/2	T

S2.2. Participants

A total of 99 participants were recruited from an online participant pool (Prolific Academic)[4], between 12-14 October 2021. The sample was restricted to participants living in the UK or the US, but no other restrictions were applied. The sample size was determined as the largest sample size that resources available at the time would allow. The survey took about 9 minutes to complete (median 9 m 9 s), and participants were compensated at a standard rate for completion. The study was approved by the PPLS Research Ethics Committee at the University of Edinburgh.

All 99 participants completed the survey and were used for analysis. Their demographic details were provided by Prolific. Participants had the option to withhold demographic data, and some demographic data had expired (were no longer available) at the time that they were downloaded; the n values below indicate the size of the sub-sample that each summary is based on. There were 46% women and 54% men (n = 68), with a mean age of 52.5, SD 15.4 (n =68); 4% were resident in the US and 96% in the UK (n = 68); and English was the first language for 96% participants (n = 68).

S2.3. Materials and procedure

The online survey, created and administered using Qualtrics,[5] was essentially identical to that reported for the Knowledge Test in the main paper, with the exception that the test encompassed 48 candidate items (Table S2.1), rather than the 30 items selected for the final scale (Table 2, main paper).

S2.4. Data processing and scoring

<u>Scoring</u>

The scoring of the Knowledge Test followed an identical procedure to that reported for the main experiment.

---

[4] http://prolific.co/

[5] http://www.qualtrics.com

Participant exclusions

From the sample of 99 participants, only 20 skipped a question, and no participant skipped more than five questions. These null responses were simply treated as missing and no participant exclusions were made.

S2.5. Analysis of item properties

To explore the item properties of the 48 items, we applied item response theory. The goal was to remove items that did not discriminate well between participants, but to retain a range of question difficulties, and topics, whilst maintaining the overall reliability of the measure. Given the binary response format, we fit all items to a 2-parameter logistic model, in which the item characteristic curves were defined by two parameters: item difficulty and discrimination. The item difficulty parameter indicated the point on the latent trait continuum at which participants have a greater than 50% chance of getting an item correct. A good test will contain items with difficulty parameters distributed across the focal range of the measure, and avoiding floor and ceiling effects. The item discrimination parameter, visually represented as the steepness of the logistic function for the item, indicates how well it discriminates between respondents at adjacent levels of the latent trait. Better items will have higher levels of discrimination. Analyses were run using the `mirt` package in R (Chalmers, 2012).

S2.6. Results and item reduction

The proportion correct and the difficulty and discrimination parameters for the 48 candidate items are listed in Table S2.2, and the item characteristic curves are shown in Figure S2.1. We removed items showing the poorest discrimination, and considered for removal items where the discrimination was weaker than other items at the same level of difficulty. At each stage of item removal, we calculated the reliable range of measurement for the scale, and the total test information to ensure that the item removal was not substantially impacting test reliability. Of the 18 items removed, 17 had discrimination values of < 0.25, resulting in flat or negative item characteristic curves; an additional item (Q38) was removed as the wording was deemed ambiguous.

The final set of 30 selected items were mostly focused on knowledge of relevant biological science (e.g. "*The reproduction number (R) of an infectious disease can change*"), with some general knowledge items (e.g. "*World War 1 had a bigger death toll than the 1918 Influenza*

Pandemic (a.k.a the Spanish Flu)", and other items testing mathematical and probabilistic reasoning ("*If I toss a coin and it lands on tails four times in a row, there is an 80% chance that it will land on heads on the next throw*"). The word "not" was subsequently removed from Q34 so that the correct answer was FALSE, in order to balance the number of TRUE and FALSE statements within the final set of 30 items (Table 2, main paper).

Table S2.2: *Proportion correct, difficulty and discrimination for the 48 candidate items. Shading indicates 18 items excluded from the final question set used in the main study.*

Item	Proportion Correct	Discrimination (a)	Difficulty (d)
Q01	0.94	2.63	4.90
Q02	0.57	0.70	0.30
Q03	0.74	0.17	1.04
Q04	0.88	0.32	2.02
Q05	0.47	-0.24	-0.10
Q06	0.96	0.57	3.31
Q07	0.69	1.17	1.05
Q08	0.69	0.41	0.82
Q09	0.71	0.50	0.93
Q10	0.73	0.51	1.08
Q11	0.17	-0.54	-1.65
Q12	0.30	0.95	-1.03
Q13	0.93	1.19	3.10
Q14	0.78	0.64	1.36
Q15	0.80	1.25	1.77
Q16	0.85	-0.27	1.74
Q17	0.73	0.83	1.12
Q18	0.34	0.24	-0.66
Q19	0.76	0.23	1.14
Q20	0.67	0.63	0.76
Q21	0.69	1.01	1.00
Q22	0.56	0.21	0.23
Q23	0.97	0.20	3.47
Q24	0.49	1.91	-0.01
Q25	0.73	1.23	1.29
Q26	0.49	0.57	-0.02
Q27	0.83	-0.12	1.57
Q28	0.69	0.00	0.79
Q29	0.41	-0.04	-0.35
Q30	0.77	0.50	1.26
Q31	0.23	0.20	-1.19
Q32	0.69	0.52	0.83
Q33	0.31	-0.47	-0.86
Q34	0.84	0.28	1.67
Q35	0.67	0.41	0.72
Q36	0.39	0.25	-0.46
Q37	0.18	0.25	-1.52
Q38	0.40	1.35	-0.60
Q39	0.40	-0.41	-0.40
Q40	0.81	1.13	1.77
Q41	0.26	0.37	-1.11
Q42	0.66	0.67	0.73
Q43	0.29	-0.19	-0.92
Q44	0.18	0.20	-1.52
Q45	0.35	0.83	-0.73
Q46	0.53	0.39	0.11
Q47	0.76	0.51	1.20
Q48	0.92	-0.46	2.51

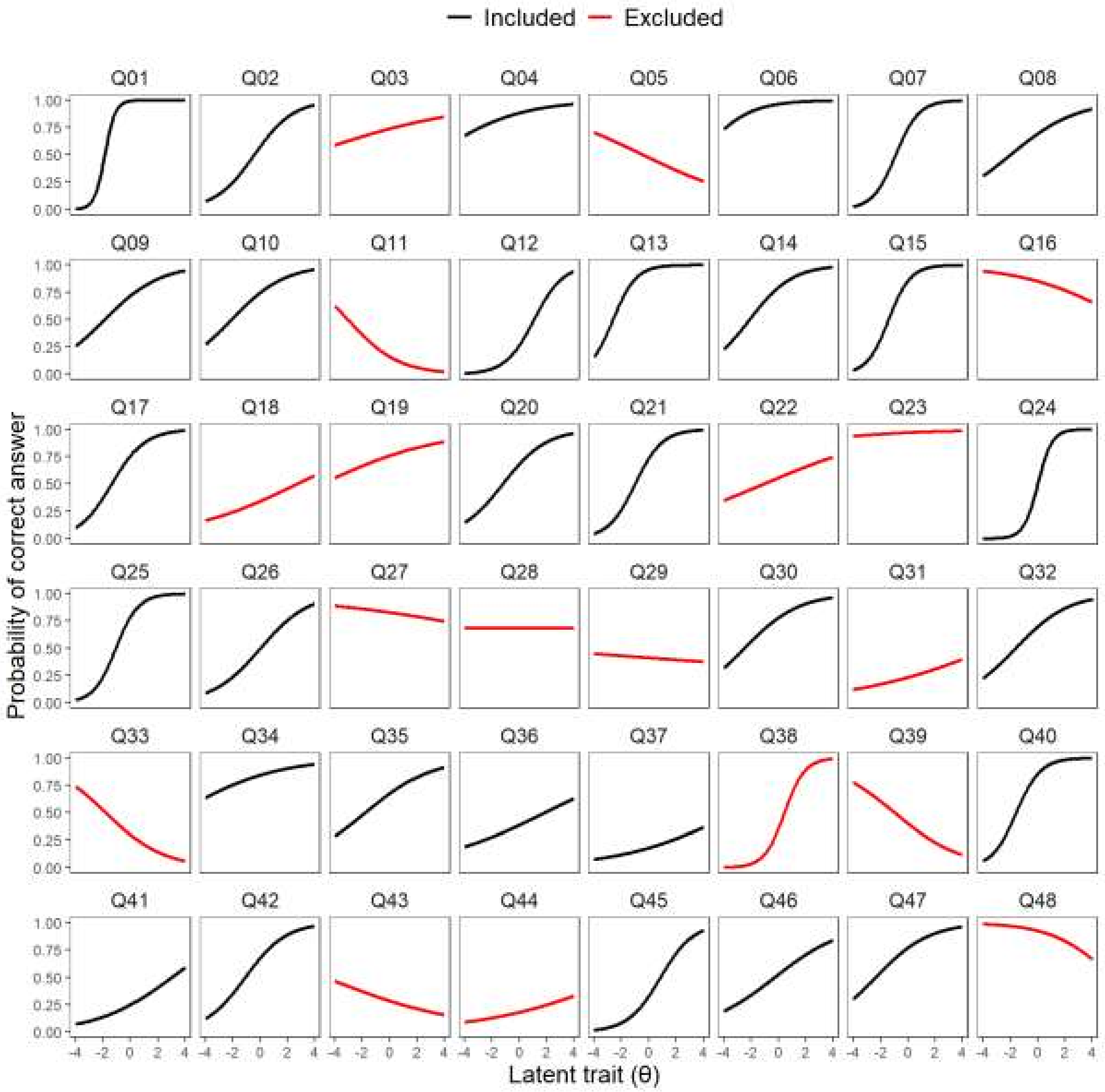

Figure S2.1: *Item characteristic curves for the 48 candidate items. The 30 items included in the final scale are distinguished from the 18 items removed.*
